# Shape variation and sex differences of the adult human mandible evaluated by geometric morphometrics

**DOI:** 10.1038/s41598-024-57617-7

**Published:** 2024-04-12

**Authors:** Aspasia Chalazoniti, Wanda Lattanzi, Demetrios J. Halazonetis

**Affiliations:** 1 Department of Prosthodontics, School of Dentistry, National and Kapodistrian University of Athens, Athens, Greece; 2 Department of Life Science and Public Health, Università Cattolica del Sacro Cuore, Rome, Italy; 3 Unit of Paediatric Neurosurgery, Fondazione Policlinico Universitario A. Gemelli IRCCS, Rome, Italy; 4 Department of Orthodontics, School of Dentistry, National and Kapodistrian University of Athens, Athens, Greece

**Keywords:** Bone, Oral anatomy, Forensic dentistry, Orthodontics

## Abstract

In cases of osseous defects, knowledge of the anatomy, and its age and sex-related variations, is essential for reconstruction of normal morphology. Here, we aimed at creating a 3D atlas of the human mandible in an adult sample using dense landmarking and geometric morphometrics. We segmented 50 male and 50 female mandibular surfaces from CBCT images (age range: 18.9–73.7 years). Nine fixed landmarks and 510 sliding semilandmarks were digitized on the mandibular surface, and then slid by minimizing bending energy against the average shape. Principal component analysis extracted the main patterns of shape variation. Sexes were compared with permutation tests and allometry was assessed by regressing on the log of the centroid size. Almost 49 percent of shape variation was described by the first three principal components. Shape variation was related to width, height and length proportions, variation of the angle between ramus and corpus, height of the coronoid process and inclination of the symphysis. Significant sex differences were detected, both in size and shape. Males were larger than females, had a higher ramus, more pronounced gonial angle, larger inter-gonial width, and more distinct antegonial notch. Accuracy of sexing based on the first two principal components in form space was 91 percent. The degree of edentulism was weakly related to mandibular shape. Age effects were not significant. The resulting atlas provides a dense description of mandibular form that can be used clinically as a guide for planning surgical reconstruction.

## Introduction

Mandibular shape has been studied extensively, both in 2-dimensional and 3-dimensional (3D) form. Changes during ontogeny involve vertical development of the ramus, increase of the gonial angle prominence, forward growth and backward inclination of the symphysis, and vertical extension of the alveolar process^1–5^. Variability within the population includes ramus and body width, the angle between the ramus and body, the height of the alveolar process and symphysis, and the inclination and width of the symphysis; sex differences, and results of aging and tooth loss, are additional sources of variation^6–11^.

In addition to its evident biological significance, understanding human mandibular form and its variations is clinically useful, particularly in cases of osseous defects resulting from trauma, pathology, congenital abnormalities, or, more frequently, tooth loss and subsequent alveolar bone resorption. Reconstruction of such defects can be guided by symmetry, if the contralateral side is present, or by employing an atlas of normative variation tailored to the remaining parts^12–16^. This atlas-based approach is commonly followed in the anthropology domain, where severely damaged specimens are reconstructed by thin plate spline (TPS) warping of a reference template^17^. A dense landmark configuration that comprehensively covers the specimen is obviously essential for detailed and accurate results.

The aims of this study were (a) to create a 3D atlas of human mandibular shape variability, using a dense landmark configuration uniformly covering the mandibular surface, which can be used for mandibular reconstruction, and (b) to explore potential sex and age differences in mandibular size and shape in an adult sample.

## Materials and Methods

### Ethical approval

All methods were carried out in accordance with relevant guidelines and regulations. The Scientific Committee of the Dental Association of Attica, Greece, and the Research Ethics Committee of the School of Dentistry, National and Kapodistrian University of Athens, gave ethical approval for this work (protocols 2151 and 109777, respectively). Informed consent was obtained from all subjects involved in the study.

### Sample

The sample consisted of cone-beam computed tomography (CBCT) images, in the form of DICOM files, from the archives of a dental imaging centre and the Department of Oral Diagnosis and Radiology of the School of Dentistry, the National and Kapodistrian University of Athens. The data had been obtained previously for diagnostic purposes, unrelated to this study and all patients had signed consent forms for use of their images for research purposes. Voxel size ranged between 0.2 and 0.4 mm. The images were anonymized, retaining only age and sex data. For this study, we excluded patients who presented with gross anatomical deformities (e.g. hemifacial microsomia, missing condyle or ramus) but included patients with normal morphological variations, missing or extracted teeth, and alveolar bone resorption due to tooth loss.

**Figure 1:**
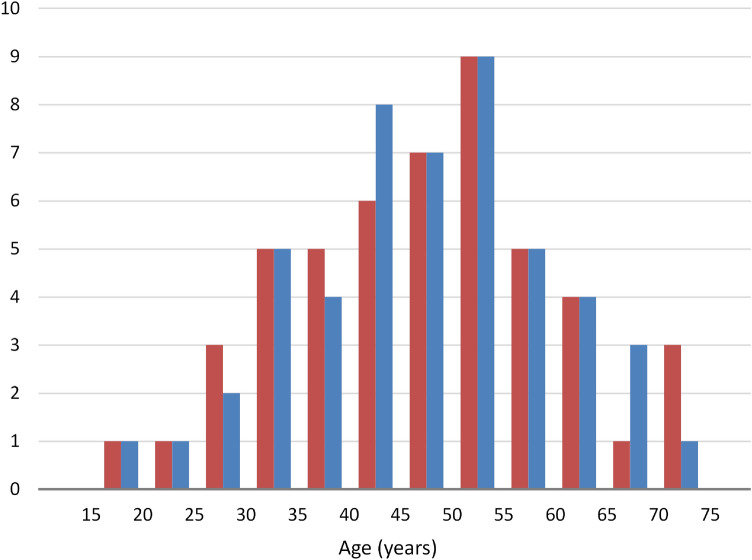
Histogram of age (years) for the female (red) and male (blue) groups.

We accrued patients until we reached 50 adult males and 50 adult females of a comparable age distribution (Table 1, Fig. 1). This size is considered sufficient for obtaining reliable data using geometric morphometric tools^18,19^. Seventy-six patients had at least one tooth missing, excluding the third molars, with an average number of 2.9 missing teeth among them (Table 1). All patients were of Greek ethnicity and sex was based on birth assignment.

**Table 1:** Demographics of the sample.

	Females	Males
Count	50	50
Age (years)		
Average (standard deviation)	46.9 (13.2)	47.3 (12.1)
Median	48.6	48.2
Range	18.9–73.7	19.1–71.0
Missing teeth		
Total teeth missing	104	115
Maximum per patient	9	11
Number of patients with missing teeth	37	39

### Preprocessing

All data processing and analysis was performed using the Viewbox 4 software (dHAL Software, Kifissia, Greece). We loaded the DICOM files and subsampled the volume by 50% for faster processing, if voxel size was 0.2 mm or smaller. The histogram of the voxel values was cropped by setting the top 0.2% of the voxels to the 99.8% value, to enhance contrast. The bone threshold was computed using Otsu multilevel histogram thresholding^20^ with three levels (air, soft tissues and bone). Segmentation was based on the computed bone threshold and manually refined at each axial slice using the software’s paintbrush, by the same observer (AC). Due to frequent artifacts from metallic restorations and prostheses, teeth could not always be segmented with confidence, and tooth surfaces should be considered unreliable. A triangular mesh surface was subsequently constructed from the segmentation using a variant of the marching cubes algorithm^21^. The internal bone structure was deleted and holes were closed, obtaining a water-tight mesh of the mandibular surface, including the dentition. Finally, a remeshing filter was applied, to increase triangle regularity and remove degenerate triangles^22^. The final meshes averaged approximately 130,000 vertices and 260,000 triangles. The average edge length of the triangles was 0.44 mm. All surfaces were uploaded and are available from the Zenodo repository (10.5281/zenodo.6335430).

### Landmarking

**Figure 2:**
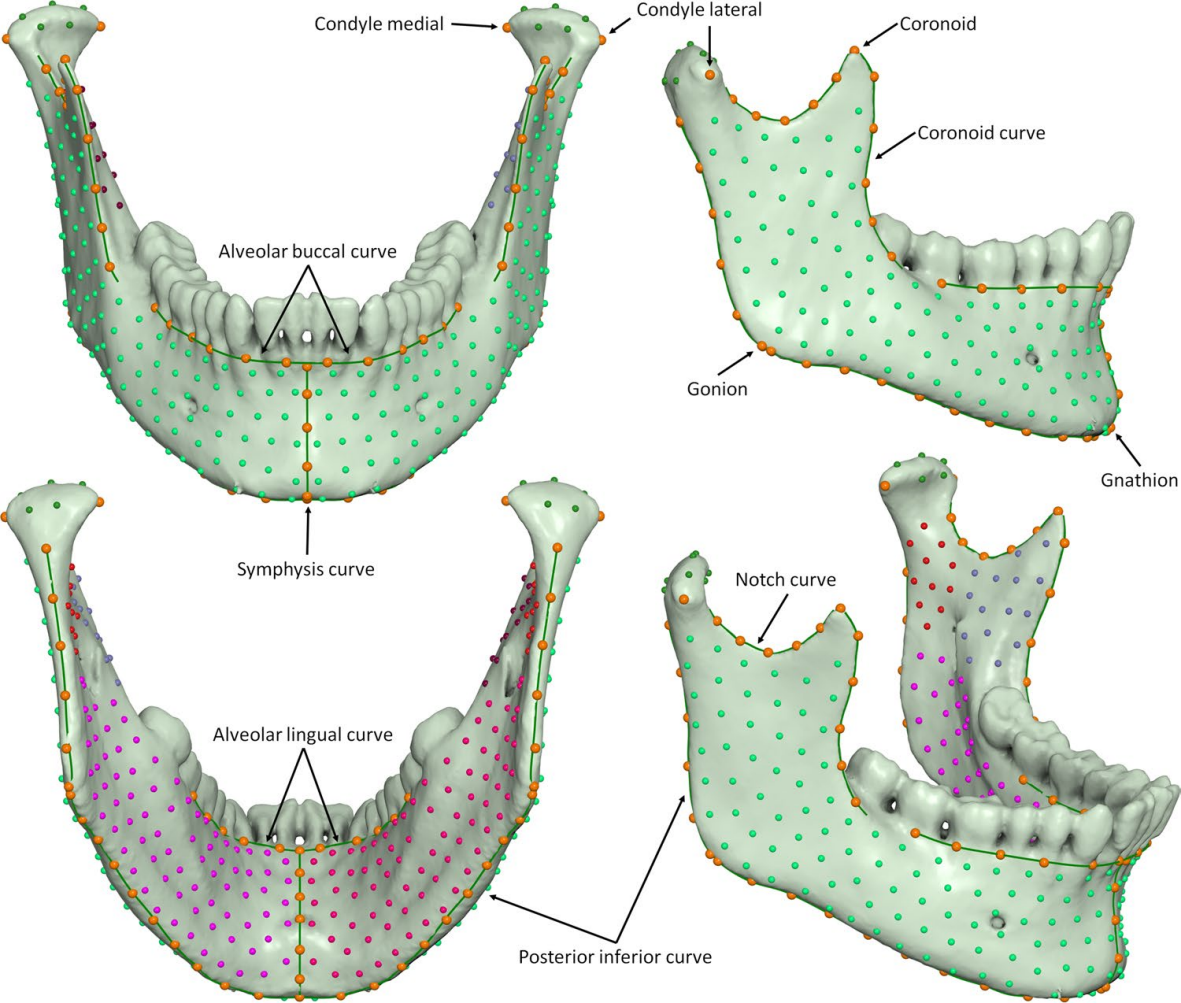
Curves and landmarks shown on one of the meshes of the sample. Surface semilandmark colours facilitate detection of gross placement errors. Green lines: curves for sliding. See Supplementary Table S1 for definition of curves and landmarks.

Digitization of the meshes was based on a template of curves and landmarks, with an even distribution over the entire mandibular surface, except for the dentition (Fig. 2, Supplementary Table S1). The template was based on a previously used template of 415 landmarks^8,23^, which we refined and augmented. We used a simplified symmetric mandibular mesh as the base for placing the landmarks. The mesh was modified by expanding it outwards by 1.5 mm, thus making it thicker overall, but most importantly in the ramus region (Supplementary Fig. S1). This avoided a common problem of the landmarks projecting on the wrong side of a thin ramus, and made ‘inflating’ the landmarks along the normals to the surface unnecessary^24^. The curves were cubic splines, adjusted to the mandibular surface by control points, and used for sliding the curve semilandmarks. There were 9 fixed landmarks, 84 curve semilandmarks and 426 surface semilandmarks, free to slide over the mesh surface, for a total of 519 points.

Landmarks Condyle lateral, Condyle medial, Gonion and Gnathion were automatically located by the software as extrema of the mesh surface along pre-specified directions (Supplementary Table S1). The condylar poles (Condyle lateral, Condyle medial) were defined as the two points farther apart from each other along the condylar axis. Recognizing that this definition is circular, we implemented an iterative procedure (Supplementary Fig. S2): first, the lateral and medial points were placed on the condyle, at the farthest and closest point from the midsagittal plane, respectively, to define an initial condylar axis. Then, each point was repositioned outwards along the condylar axis and re-projected to the closest point on the mesh. Due to the ellipsoid shape of the condylar head, the projections fall closer to the true poles and define a new condylar axis. A few iterations of this step were sufficient to stabilize the points at the medial and lateral poles, at the farthest distance between them.

The curves Notch, Coronoid, and Posterior-inferior were also located automatically based on mesh curvature data and extremal directions. The procedure was similar to that used for the condylar poles, but the direction of outward movement was fixed (superior, inferior or posterior, depending on the curve). The Alveolar buccal and Alveolar lingual curves were digitized manually to follow the alveolar bone crest. All other landmarks were initially placed by a thin plate spline (TPS) warping of the template to the previously digitized points and curves, and then projected on the closest point of the mesh surface. To easily detect projection errors at this step and subsequent sliding steps, the surface semilandmarks were coloured differently, based on their anatomical position (Supplementary Fig. S1 and Fig. 2). Digitization was inspected and corrected manually. All digitizations were performed by the same investigator (AC).

### Geometric morphometrics

Shape analysis was based on the traditional toolbox of 3D geometric morphometric (GM) methods. After each mesh was digitized, the semilandmarks were allowed to slide against the sample average shape, to minimize bending energy^25–27^, then all landmarks were re-projected on their corresponding curves or on the mesh surface. Sliding and projecting was repeated five times, each time reducing the sliding step in a linear fashion, to avoid oscillations in landmark position. The average shape was then re-computed and used as the reference configuration for a subsequent iteration of sliding and projecting. Finally, we aligned all configurations using the generalized Procrustes alignment (GPA) method^28^, computed centroid size, and ran a principal component analysis (PCA) to extract the most significant shape variation patterns as principal components (PC). PCA was performed both in shape space and form space, which includes the logarithm of the centroid size—ln(CS)—as an extra variable^17^. The final landmark coordinates are available from Zenodo (10.5281/zenodo.6335430).

The region of the gonial angle has been reported to differ between the sexes, apparently due to stronger muscle attachment in males, causing gonial eversion and ramus flexure^29,30^. In order to investigate potential sex differences in this area, we ran a regional GPA and PCA analysis, confined to the landmarks of the ramus, on the right side. This included a total of 123 landmarks (Supplementary Fig. S3).

Additionally, we computed the volume enclosed by the mesh surface, and calculated the mesh normalized centroid size as the square root of the average sum of the squared distances of the mesh vertices to their centroid. Interpretation of these measurements needs caution, as the meshes also included the teeth (see “Discussion” section).

### Statistical analysis

Digitization error was tested by repeating the digitization of 20 randomly selected meshes by a second investigator (DJH). We assessed measurement error by comparing the Procrustes distances between repeats to the extent of the sample in shape space and also by a Procrustes ANOVA^31,32^.

Shape patterns were visualized by TPS warping the average shape along the PC directions over the range of ± 3 standard deviations (SD). The distribution of the sample was inspected in shape space by a 3D plot of the relevant PCs. Shape comparison between the sexes was based on the Procrustes distance between the group means, and on Goodall’s F test on the squared Procrustes distances, both using permutation tests (100,000 permutations without replacement). A multivariate general linear model (MANOVA) was run, with PCs as the dependent variables, sex as the fixed factor, and age, centroid size, volume, and number of missing teeth as covariates. The model was then adjusted based on the interaction effects and multicollinearity.

Sex shape differences were visualized by TPS warping of the average shape along the trajectory connecting the average male and female shapes, exaggerated for clarity by three times the Procrustes distance between the sexes in shape space. Linear discriminant analysis (LDA), with a leave-one-out cross-validation (jackknifing) procedure, was used to compute the percentage of correctly classified subjects by sex, based on shape and size. For LDA and MANOVA, we used the non-trivial PCs, computed as those for which both the ‘random average under permutation’ (Avg-Rnd) and ‘eigenvalue under randomization’ (Rnd-Lambda) stopping criteria were satisfied (100 permutations)^33^. We tested static allometry by regression of the ln(CS) on the shape PCs. Sex differences in mandibular size were also tested by comparing the mandibular volumes and mesh centroid sizes.

Geometric morphometric tests (including PCA stopping criteria) and visualizations were performed by Viewbox (dHAL Software, Kifissia, Greece); other statistical tests were performed using MorphoJ version 1.08.01^31^, StatsDirect 1.8.10 (StatsDirect Ltd, Wirral, UK), PAST 4.09^34^ and IBM SPSS Statistics for Windows, version 28.0 (IBM Corp., Armonk, NY, USA).

## Results

### Digitization error

The average Procrustes distance between the 20 repeated digitizations was 2.9% of the extent of the sample in shape space, as determined by double the distance of the farthest specimen from the centre of the sample (diameter of hypersphere enclosing the sample). The repeated digitizations virtually coincided with the originals in the PC1-PC2 plot (Supplementary Fig. S4). Based on the Procrustes ANOVA (Supplementary Table S2), digitization error explained 0.4% of the total variation and repeatability was 0.992^32^.

### Shape analysis

**Figure 3:**
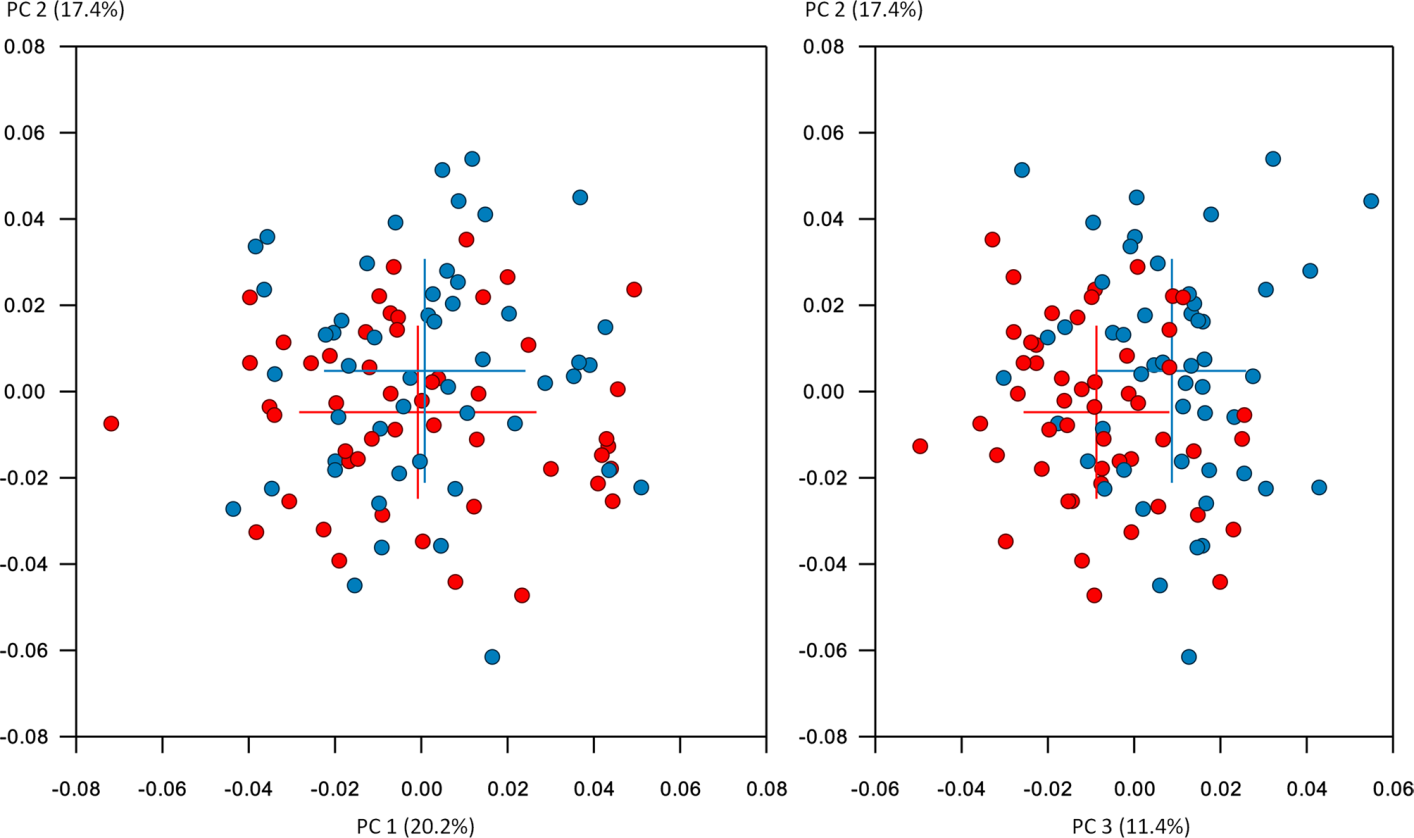
The sample plotted in shape space. Red: females, blue: males. Crosses show average of each group and standard deviations.

The plot of the sample in shape space is shown in Fig. 3 and the plot in form space in Supplementary Fig. S5. The Avg-Rnd and Rnd-Lambda stopping criteria^33^ showed 12 non-trivial PCs for the shape space analysis and 13 PCs for the form space analysis. In shape space, the first 3 PCs described 49% of the shape variance; the first 12 PCs described 80% of the variance (Supplementary Table S3).

**Figure 4:**
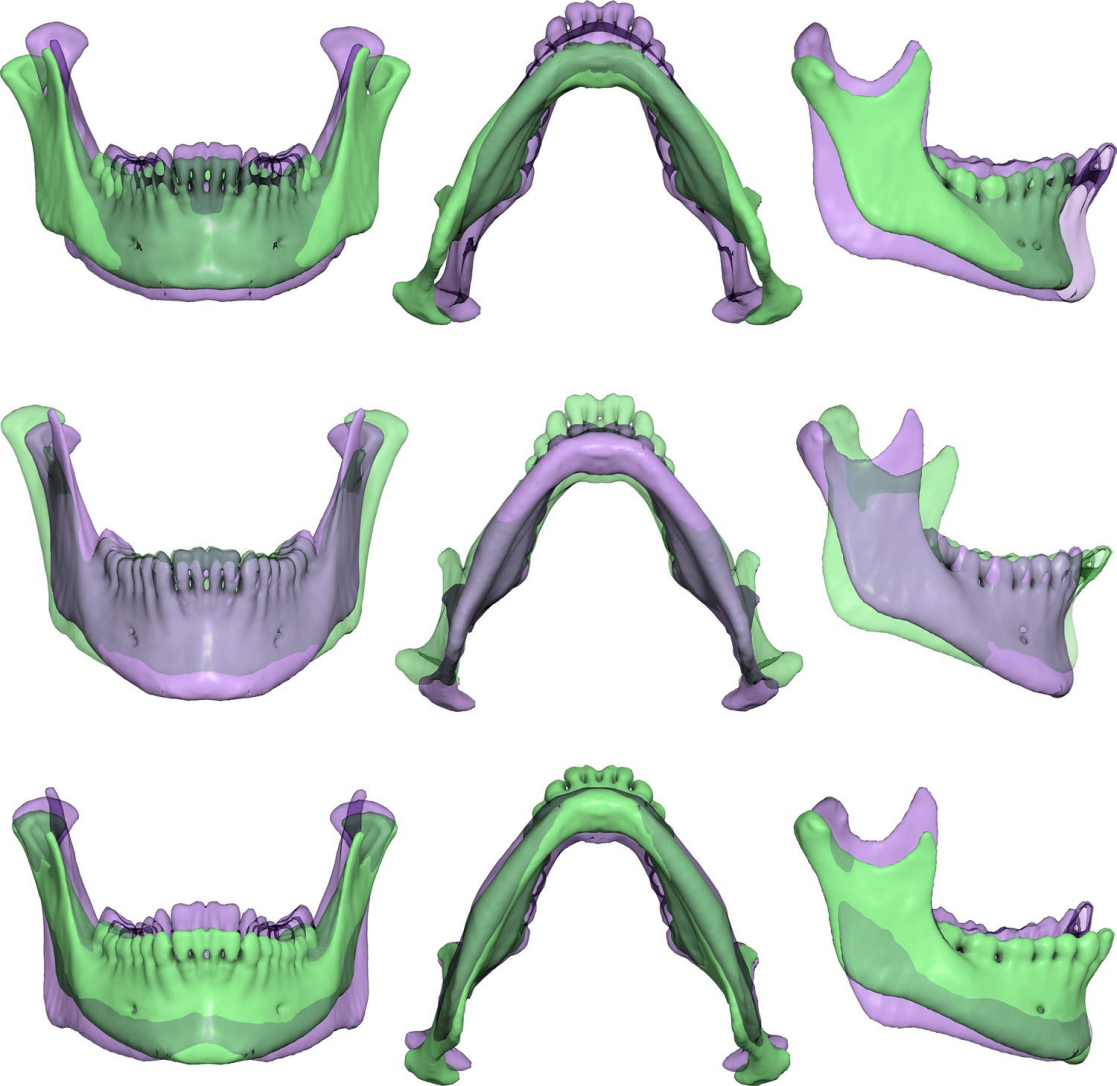
Shape variations described by the first 3 PCs. Average shape warped along each PC at − 3 and + 3 standard deviations (green and purple, respectively). Top row: PC1, middle row: PC2, bottom row: PC3.

Shape variation, as described by the first 3 PCs of shape space, is shown in Fig. 4 (average shape warped along each PC at − 3 and + 3 standard deviations). PC1 described variability in the width of the mandible, the height of the ramus and the alveolar process. PC2 described variability in the angle between the ramus and the corpus, whereas PC3 described variability in the height of the coronoid process, the inclination of the symphysis and the prominence of the mandibular angle.

### Univariate correlations

Table 2 shows correlations between the variables sex, age, ln(CS), number of missing teeth and mesh volume. Sex was related to landmark centroid size and mesh volume, and age was related to the number of missing teeth. There was no correlation between sex and the number of missing teeth. Volume and number of missing teeth showed a low correlation, which is not significant if a Bonferroni correction is applied to Table 2.

**Table 2:** Correlation matrix of independent variables.

	ln(CS)	Age	#Missing teeth	Mesh volume
Sex	− 0.801 (< 0.001**)	− 0.012 (0.902)	− 0.055 (0.588)	− 0.523 (< 0.001**)
ln(CS)		0.048 (0.632)	0.034 (0.739)	0.672 (< 0.001**)
Age			0.395 (< 0.001**)	− 0.030 (0.764)
#Missing teeth				− 0.251 (0.012*)

Pearson’s correlation coefficient (P value).

Sex coded as: 1: female; 0: male.

*P < 0.05; ** P < 0.01.

The correlation between volume and ln(CS) was high for the whole sample (r^2^ = 0.45), and lower for each group separately (females: r^2^ = 0.19, P = 0.0016; males: r^2^ = 0.28, P < 0.0001). A higher correlation might be expected, but the volume effect of tooth loss and ensuing alveolar bone resorption is primarily in the vertical dimension, without significantly influencing the average distance of the landmarks from the centroid, which is located midsagittaly between the alveolar processes.

### Sex differences

**Figure 5:**
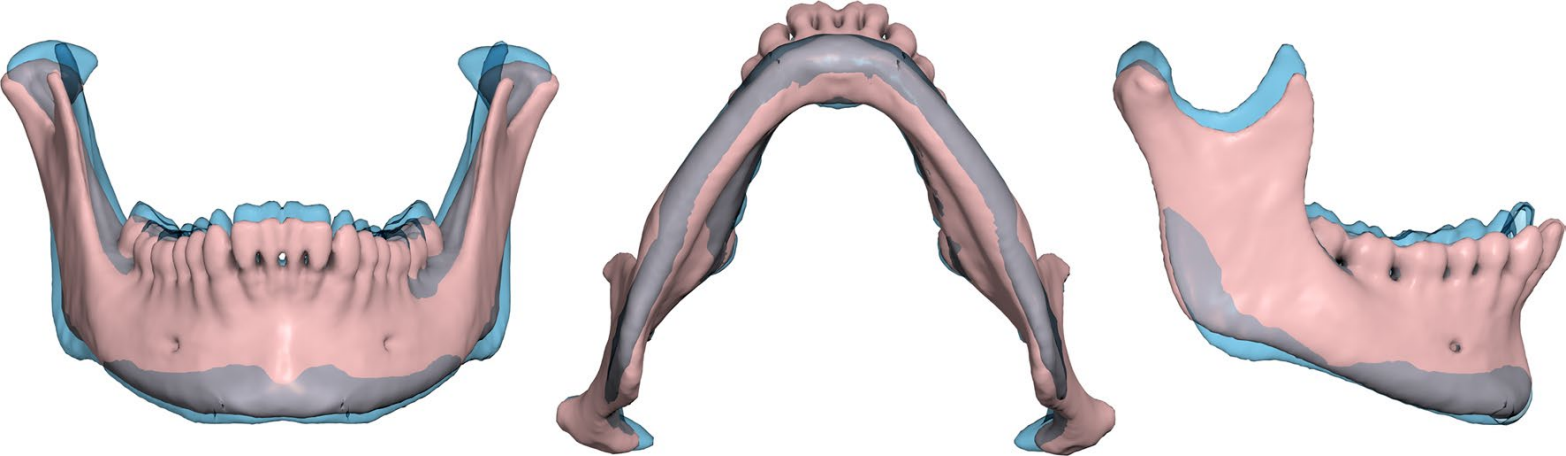
Superimposition of the male and female shapes. To enhance differences, the average male and female shapes were moved apart in shape space by three times the original Procrustes distance between them, along the male–female shape vector. Both shapes are scaled to the same centroid size. Male: blue, female: red.

The male and female groups differed significantly in shape space (permutation tests; Procrustes distance between means: P < 0.0002; Goodall’s F test: P < 0.0002), mainly along PC2 and PC3. Figure 5 shows the intergroup shape difference of the average male and female shapes, exaggerated 3 times along the male–female vector, keeping the same centroid size. Males showed a wider mandible at the gonial angles, but narrower at the condyles and coronoids, a higher ramus with higher condylar and coronoid processes, more pronounced antegonial notch, and a more posteriorly inclined symphysis with prominence at menton. In contrast, females had a larger mandibular angle, a wider ramus and a more gracile body. These are relative differences, with size adjusted to be equal between the two groups.

**Figure 6:**
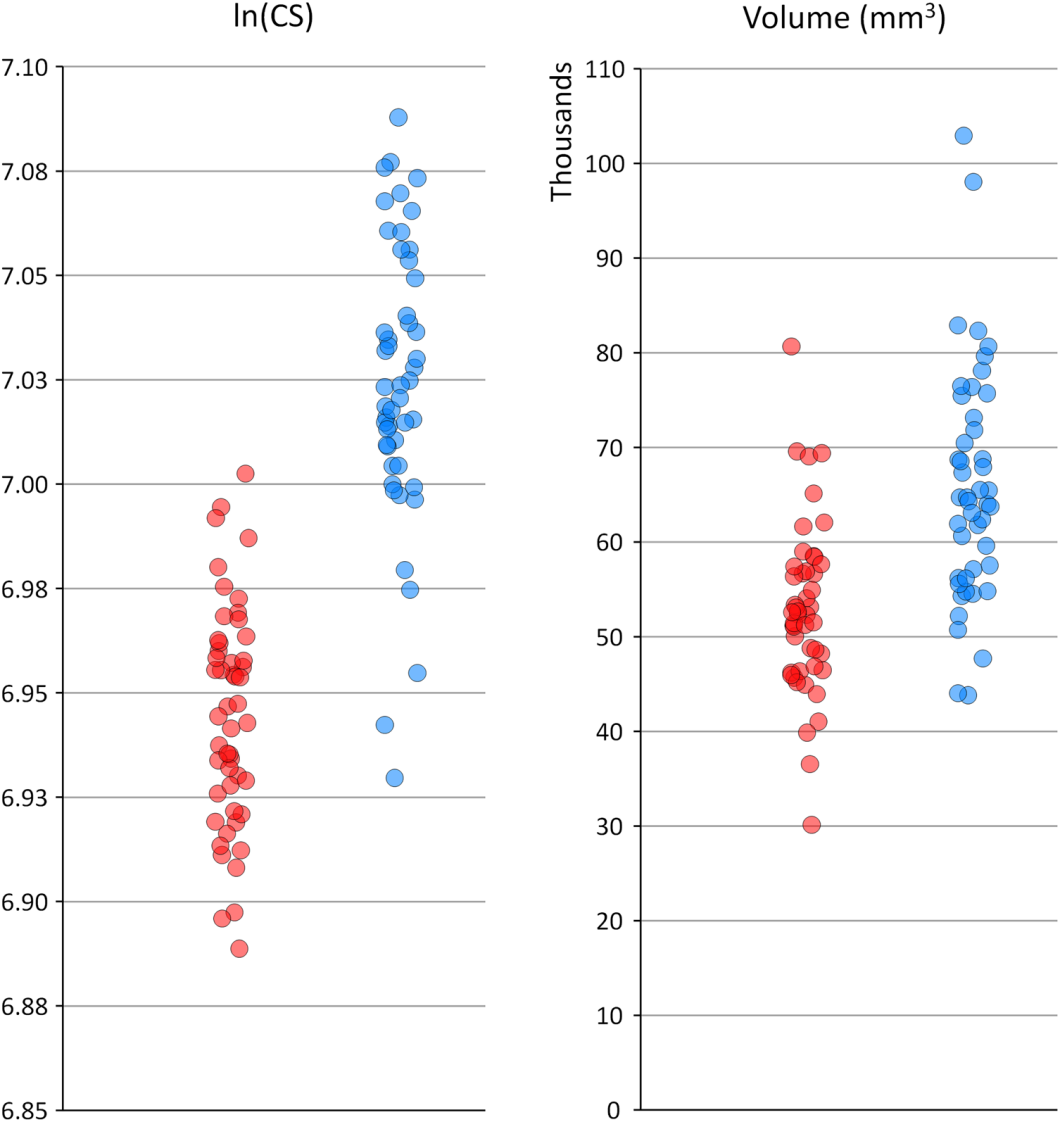
Jitter strip plots of ln(CS) and mesh volume for the female (red) and male (blue) groups.

There was a clear sex difference in mandibular size, expressed as the mesh centroid size (males larger by 8.9%), the mandibular volume (25%), or the landmark centroid size (8.3%) (Table 3, Fig. 6). The plot of the sample in form space showed very little overlap (Supplementary Fig. S5). A multivariate general linear model (MANOVA) of the first 12 PCs in shape space, as dependent variables, showed that sex and number of missing teeth were significant factors (Table 4).

**Table 3:** Size variables. Student’s t-test.

	Females	Males	t-test
Mesh centroid size (mm)			
Mean (SD)	45.50 (1.43)	49.56 (1.96)	t = 11.8, P < 0.0001
Range	42.45–49.06	44.26–53.17	
Mandibular volume (mm^3^)			
Mean (SD)	52,918 (9180)	66,133 (12,360)	t = 6.0, P < 0.0001
Range	30,121–80,683	43,834–102,954	
Landmark ln(CS)			
Mean (SD)	6.94 (0.03)	7.02 (0.03)	t = 13.2, P < 0.0001
Range	6.89–7.00	6.93–7.09

**Table 4:** General multivariate linear model of PCs 1-12 in shape space, by sex and number of missing teeth.

Effect		Value	F	Degrees of freedom	P
Intercept	Pillai’s Trace	0.189	1.668	12, 86	0.088
	Wilks’ Lambda	0.811	1.668	12, 86	0.088
Sex	Pillai’s Trace	0.383	4.456	12, 86	< 0.001
	Wilks’ Lambda	0.617	4.456	12, 86	< 0.001
#Missing teeth	Pillai’s Trace	0.300	3.066	12, 86	0.001
	Wilks’ Lambda	0.700	3.066	12, 86	0.001

Discriminant analysis using the first 4 principal components in form space (where PC1 is heavily weighted on centroid size) showed a 93% correct sex classification. This reduced to 91% when including only PC1 and PC2 and did not increase beyond 93% even by including all 13 non-trivial PCs. LDA using the PCs of shape space had a correct classification of 59% when including the first 2 PCs, increasing to within the 66–72% range when adding PCs 3–12 (Supplementary Table S4).

### Static allometry

Regression of ln(CS) on the shape variables of the pooled sample showed a significant but low correlation, less than 5% of the shape variance explained by size (10,000 permutations, predicted variance = 4.44%, P < 0.0001). However, this was lost when regressing each group separately (Table 5). In contrast, mandibular volume was related to the shape variables in both groups separately, and in the pooled sample, explaining almost 13% of shape variance in the female group. The number of missing teeth was also related to shape, but only in the male group and the pooled sample (Table 5).

**Table 5:** Correlations between independent variables and shape variables (PCs in shape space), tested with 10,000 permutations.

	Pooled sample		Female group		Male group	
	R^2^	P value	R^2^	P value	R^2^	P value
ln(CS)—shape	4.44	0.0000*	2.63	0.2055	1.58	0.6650
Age—shape	1.41	0.1584	1.97	0.4513	3.18	0.0956
Volume—shape	8.71	0.0000*	12.59	0.0000*	10.26	0.0000*
# Missing teeth—shape	3.71	0.0001*	3.02	0.1234	5.81	0.0012*

*P < 0.01.

### Age

There was no statistically significant correlation between age and shape, or between age and centroid size (Table 5 and Table 2, respectively).

### Ramus analysis

**Figure 7:**
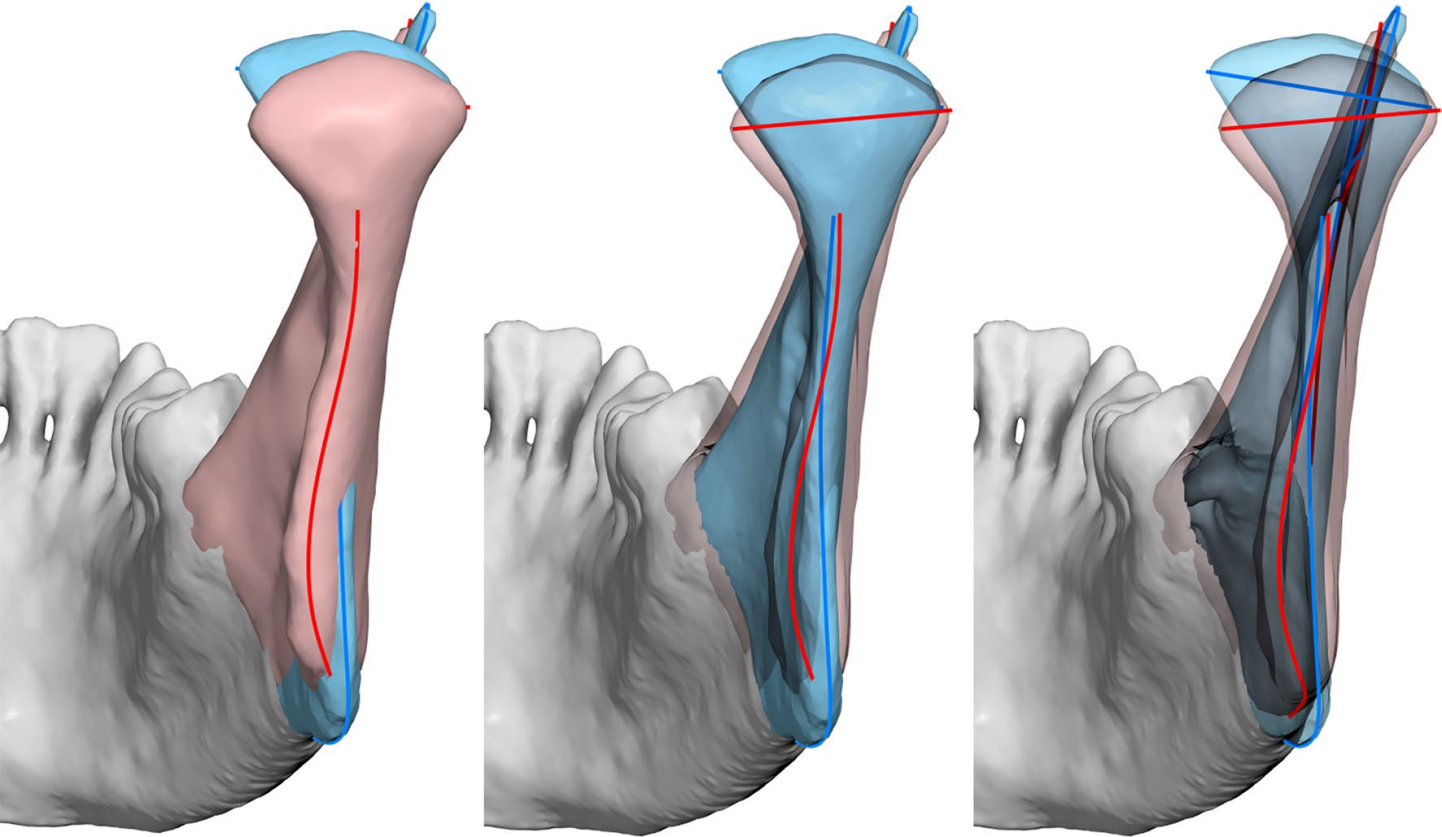
Superimposition of the male (blue) and female (red) groups showing shape differences of the ramus (here exaggerated). Middle: female ramus transparent; right: both rami transparent. Note female outline (red) curving towards the lingual side, and difference in condylar axis angulation.

As expected, a clear size and shape difference was also observed for the isolated ramus. Centroid size of males was larger by 11.8% (t-test, P < 0.0001). Shape differed significantly (P = 0.0003, 10,000 permutations). Similar shape differences were noted to those observed for the whole mandible (Fig. 7). We could not observe gonial angle eversion in the male group, nor ramus flexure.

## Discussion

The morphology of the mandible does not provide a large number of distinctly identifiable landmarks to attain comprehensive coverage of its surface, potentially leading to loss of important phenotypic information. However, even if achievable, dense landmarking would not be sufficient unless complemented with reasonable confidence of landmark homology (correspondence) across specimens. We recognize two main approaches for establishing correspondences and landmarking of 3D surface meshes; both use a reference template (atlas) with the landmarks of interest already identified on it. The first approach performs a rigid alignment of the template mesh to match the target, followed by deformable registration to refine the match, and then transfers the landmarks from the atlas to the target mesh. Examples are ALPACA^35,36^ and MeshMonk^37,38^, which mainly differ in their non-rigid registration algorithm. Variants of this approach abolish landmarks altogether and achieve correspondences between the vertices of the meshes directly^14,39^.

The second approach needs digitization of a (relatively small) subset of the landmarks on both meshes, then performs a TPS warping of the template, driven by this subset of landmarks, and transfers the remaining points to the target mesh, usually by projection on the mesh surface. Thus, the first approach is essentially a mesh-to-mesh registration, whereas the second is a TPS warping of point configurations. This method belongs to the geometric morphometric toolbox^17,28,40^ and is usually followed by sliding of semilandmarks to enhance geometric correspondence^25,26^ and mapping of the specimens in a shape space via generalized Procrustes alignment^41^ and PCA.

The shape space is the final goal of all methods, as it represents a statistical shape model^13,14^ which describes population variability and can be used both as a reference for testing novel shapes and as a generative source for creating plausible anatomy. We consider GM methods preferable, due to their solid statistical foundation and excellent visualization tools. Although highly dense models are produced from methods that establish correspondences at the mesh vertex level^13,14,39^, the anatomical correspondence (homology) is not guaranteed^26,42^.

Studies of the mandible with dense landmarking are scarce. Most studies limit the landmarks on the anterior and posterior ramus ridges, the mandibular notch, the inferior outline, and the symphysis outline on the midsagittal plane, in addition to ubiquitous conventional landmarks, such as Gonion, Gnathion, Coronoid and the condylar poles^5–7,9,43–46^. The total number of landmarks ranges from below 20 (e.g.^45^) to above 100 (e.g. 113^44^, 301^46^ or 1000^5^); however, seldom is the mandibular surface between ridges landmarked^5,8,23,36^.

In creating an atlas of shape variability, the design of the template is a key factor^24^. The base mesh does not need to be detailed or be one of the meshes of the sample; indeed, simpler geometries sometimes work better^24^. We used a simplified symmetric mandibular mesh, expanded outwards by 1.5 mm, to avoid the common problem of the landmarks projecting on the wrong surface^24^, especially in the area of the gonial angle, where the ramus can be thin and the gonial angle everted. We aimed for a large number of landmarks, dispersed evenly over the whole surface, to capture both the shape of the main ridges and the smooth areas in-between. The number of fixed landmarks was small, limited to the condyle poles, the coronoid processes, Gonion and Gnathion. Gonion is a problematic landmark, showing high identification error, both in 2D and 3D digitizations^47–49^. Although we could set this as a sliding semilandmark, or remove it altogether, we opted to retain it as a sentinel between the ramus and the corpus, to avoid the curve and surface semilandmarks from invading the wrong area. However, we located it algorithmically using a clearly defined geometric procedure, to reduce identification error (Supplementary Table S1). Gnathion was similarly located, as the farthest point from the condyle on the midsagittal plane. Locating landmarks algorithmically avoids subjectivity, and ensures repeatability and validity, although biological homology is debatable^42^. The curve semilandmarks were few and sparsely dispersed, to allow them to adjust by sliding, since a very high density effectively prohibits sliding and reverts to equidistant sampling. The surface semilandmarks were dispersed on the template mesh via a diffusion algorithm, to ensure an initially even distribution.

Our sample was a convenience sample, imaged for various reasons, most commonly for dental implant planning and third molar pre-extraction evaluation. Although there is no assurance that the average coincides with the average of the population, research has shown minimal effect of including even extreme cases^50^. Sample size was adequate for assessing average shape and shape variability^18,19^. Sex grouping was based on birth-assignment. Although several factors can affect the phenotype, such as genetic variations, sex chromosomes, epigenetic variation, hormones, environmental factors, and others^51^, it was not possible to investigate them in the present sample.

Landmarking was accurate, as shown by the repeated digitizations, because the curves were placed on well-defined ridges and the remaining surface points were located by a TPS warping of the template followed by sliding. Fixed landmarks were placed by automated heuristics (e.g. Gnathion, Gonion) further minimizing subjective identification^52^. The only curves that required full human intervention were the alveolar buccal and lingual curves.

Almost 80% of the shape variance was described by the first 12 PCs, the first 3 of these describing 49%, so most of the shape variability was included in only a few PCs, comparable to previous work^7,36^. Van der Wel et al.^11^ report a larger spread of shape variance among the PCs; this can be attributed to their sample comprising patients treated by orthognathic surgery, therefore potentially more extreme cases, and to segmentation artifacts in the area of the teeth, due to metallic orthodontic appliances. The number of landmarks is a significant factor affecting the percentage of shape variance distributed between the PCs. Studies with a few landmarks report a large fraction of variance in the first few PCs because shape is more coarsely measured (e.g. 14 landmarks: 67% shape variance in the first 2 PCs^53^, 13 landmarks: 61% variance in the first 2 PCs^9,45^).

The shape patterns were similar to those reported elsewhere, mainly related to mandibular width, angulation between the ramus and corpus, inclination of the symphysis and prominence of the gonial angle. PC1 described mandibular width variation, in relation to ramus height and corpus length (Fig. 4) whereas PC2 mainly described the ramus-corpus angulation. The same primary patterns are seen in the work of van der Wel et al.^11^, and potentially Fournier et al.^36^ and Kim et al.^39^, although the visualizations in those publications do not facilitate a direct comparison. The shape patterns obviously depend on several factors, including ethnicity, age, sex, and degree of edentulism. Our sample was mono-ethnic, equally divided by sex, and of low edentulism prevalence (average number of teeth missing: 2.2, Table 1), so the results need to be assessed under these conditions.

A significant effect of edentulism on mandibular shape has been observed^9,45,54^, which we detected here, but only in the male group and the pooled sample. In addition to reduction in the height of the alveolar process, loss of teeth was associated with retraction of the anterior alveolar area with relative prominence of menton, increase of the gonial angle and intercondylar distance, and posterior inclination of the ramus (Supplementary Fig. S6). We mention these associations with great caution, even though they agree with previous reports overall^9,45,54^, since our sample contained very few patients with many (> 5) missing teeth, did not contain completely edentulous mandibles, and alveolar resorption had not progressed significantly in several patients.

A clear sex difference was evident, both regarding size and shape, as expected for an adult sample^10^. Centroid size, computed from the mesh vertices, was 8.9% larger in males; however, mesh volume was 25% larger. A discrepancy between the two is expected because, with scaling, volume increases to the third power, whereas centroid size to the first power. However, the expected volume change would be larger, at 29% (1.089^3^ = 1.291). This can be explained by the mandible’s shape and the position of the centroid, which lies in empty space, on the midsagittal plane, at the level of the molars. The distance of the landmarks relative to the centroid is affected mostly by variation in mandibular width, and not so much by variation in ramus height, ramus anteroposterior width, or ramus and corpus thickness, factors that significantly affect volume. The superimposition of the size-adjusted averages (Fig. 5) shows differences in shape that explain this discrepancy between volume and centroid size ratios, e.g. a higher ramus and a more pronounced gonial and symphyseal area in the males.

The mesh-based size and volume measurements included the tooth regions. Any sex differences assessed by these variables could therefore be confounded by an unequal number of missing teeth between the groups, or by sex differences in tooth size. The first factor was not pertinent here because the degree of edentulism was similar between the groups (Table 1), but the second factor could be relevant, as males tend to have larger teeth than females^55,56^. However, the dentition area is small relative to the whole mesh, and tooth-size sex differences are also too small for them to be of concern here. In any case, these measurements are of low reliability, additionally because volume segmentation was often uncertain due to streaking artefacts.

The landmark-based centroid size difference was similar to the mesh-based difference (8.3% vs. 8.9%, respectively) and is considered more reliable as it is not affected by inclusion of teeth. Size differences have been noted in all previous studies of adult samples. Franklin et al.^7^ reported almost the same centroid size difference (7.8%) for their sample of 30 mandibles. Vallabh et al.^57^ list various linear measurements, of which ramus height shows the largest relative difference between sexes (14%) whereas width measurements are comparable to our centroid size ratio (intercondylar width: 5.6% and intergonial width 8.7%). This difference in intercondylar and intergonial widths is also reflected in the shape difference we detected (Fig. 5). Kranioti et al.^58^, in a sample of the same ethnic origin as ours, report a comparable inter-gonial width difference of 7.6%, giving a sex classification accuracy of 71%.

Shape differences were less pronounced than size differences, as seen when comparing Fig. 3 and Supplementary Fig. S5. In addition to a higher ramus, more pronounced gonial and mental areas, males showed a wider inter-gonial distance. Such sex differences have been noted by other investigators as well^7,8,53^.

One of the traits considered a male characteristic is gonial eversion, presumably arising from a strong masseteric attachment. However, evidence suggests that this difference is lower than initially assumed^29,59^. To overcome the qualitative nature of this trait, Oettlé et al.^59^ applied GM methods confined to the posterior ramal and gonial areas and obtained quantitative data. Although they detected differences between males and females, mainly in the extent and location of the eversion, the accuracy of sexing was below 75%. Our sample showed a larger inter-gonial width in males (Fig. 5), but there was no clear gonial eversion when examining the gonial area. On the contrary, the female outline curved towards the lingual and the male outline was straight (Fig. 7). A difference in condylar axis angulation was also observed.

Another alleged dimorphic trait is ramus flexure, an “angulation of the posterior border of the mandibular ramus at the level of the occlusal surface of the molars”^30^. Although the initial results for this trait were positive, later evidence is conflicting^10,29,60,61^. Unfortunately, this trait is qualitative and could not be incorporated into the GM analysis. Visual inspection of the posterior ramus border did not show flexure in our sample (Fig. 7).

Discriminant analysis based on the first 2–4 PCs in form space was very successful in assigning subjects to their correct group (91–93% accuracy). However, this was achieved mainly due to size differences, similarly to previous research using linear measurements between anatomical landmarks^10,58^. Classification accuracy was higher than that reported by previous studies using conventional linear and angular measurements. Kranioti et al.^58^ report an accuracy of 80% based on 2 linear variables in a Greek population. Other reports vary, from around 75% to almost 90%, using univariate or multivariate models^62–69^. Franklin et al.^70^ report an exceptionally high accuracy of 95%, but this needs to be interpreted with caution, as 10 variables were applied on a sample of 40 mandibles, suggesting a danger of overfitting.

We did not detect an age-related shape change, even though the degree of edentulism was related to both age and shape. This is in contrast to some previous reports, e.g., Costa-Mendes et al.^53^. As noted above, our sample size was relatively small in the higher and lower age bins; however, we had a similar degree of edentulism in both sex groups, whereas this was not recorded in^53^ and could have biased the results.

The atlas (average form and variability patterns) can be used clinically as a guide for planning surgical mandibular reconstruction. In cases of missing or deformed parts, the intact mandible can be used to fit the model and obtain a plausible anatomical form of the remaining. Virtual reconstruction based on statistical shape models has been demonstrated with good results^12,15,16^. It is evident that dense landmarking, for detailed representation of the anatomy, and matching of the population from which the model was constructed to the patient characteristics, are essential factors of success.

### Limitations

Future research on mandibular shape variability and sex differences could improve on this study in several ways. First, the template could be augmented with more points on the condylar head, so variability in condylar form could be investigated in detail. However, a GM analysis, focused on that region, instead of encompassing the whole mandible, would probably be more appropriate. Such an analysis could be performed on the mental region, as well, as this has also been reported to show sex differences. A notable challenge with regional analyses lies in delineating the region to be studied, particularly when clear morphological boundaries are lacking.

A second limitation was related to sample composition. Ours was of Greek ethnicity, and contained few patients in the tail ends of the age distribution, making it difficult to evaluate age effects. Also, time of tooth loss was not available. The effect of tooth loss on alveolar bone resorption, and subsequent functional issues that depend on prosthetic rehabilitation are expected to affect mandibular shape, but could not be evaluated here. Our analysis showed that tooth loss and sex were the main factors related to mandibular shape; since tooth loss is heavily tied to age, it is not easy to uncover a potential age-related effect of sex. A sample with minimal edentulism over a wide age range would be valuable in this respect.

### Supplementary Information

Supplementary Information.

## Data Availability

The mandibular meshes used in this study, the Viewbox template, and the landmark coordinates of each mandible in the sample, are available from the Zenodo repository (10.5281/zenodo.6335430).

## References

[1] Björk A, Skieller V (1983). Normal and abnormal growth of the mandible. A synthesis of longitudinal cephalometric implant studies over a period of 25 years. Eur. J. Orthod..

[2] Enlow, D. H. Growth of the mandible. In *Essentials of Facial Growth, Second Edition* (eds. Enlow, D. H. & Hans, M. G.) 63–90 (Needham Press Inc., 2008).

[3] Coquerelle M, Bookstein FL, Braga J, Halazonetis DJ, Weber GW (2010). Fetal and infant growth patterns of the mandibular symphysis in modern humans and chimpanzees (Pan troglodytes). J. Anat..

[4] Klop C, MAGIC Amsterdam (2021). A three-dimensional statistical shape model of the growing mandible. Sci. Rep..

[5] Remy F (2019). Morphometric characterization of the very young child mandibular growth pattern: What happen before and after the deciduous dentition development?. Am. J. Phys. Anthropol..

[6] Nicholson E, Harvati K (2006). Quantitative analysis of human mandibular shape using three-dimensional geometric morphometrics. Am. J. Phys. Anthropol..

[7] Franklin D, O'Higgins P, Oxnard CE, Dadour I (2007). Sexual dimorphism and population variation in the adult mandible: Forensic applications of geometric morphometrics. Forensic Sci. Med. Pathol..

[8] Coquerelle M (2011). Sexual dimorphism of the human mandible and its association with dental development. Am. J. Phys. Anthropol..

[9] Guevara-Perez SV, Behr M, Thollon L (2019). Exploratory study of the three-dimensional morphological variation of the jaw associated to teeth loss. J. Stomatol. Oral Maxillofac. Surg..

[10] Hazari P, Hazari RS, Mishra SK, Agrawal S, Yadav M (2016). Is there enough evidence so that mandible can be used as a tool for sex dimorphism? A systematic review. J. Forensic Dent. Sci..

[11] van der Wel H (2023). Morphological variation of the mandible in the orthognathic population-a morphological study using statistical shape modelling. J. Pers. Med..

[12] Raith S (2017). Planning of mandibular reconstructions based on statistical shape models. Int. J. Comput. Assist. Radiol. Surg..

[13] Ambellan F, Lamecker H, von Tycowicz C, Zachow S (2019). Statistical shape models: Understanding and mastering variation in anatomy. Adv. Exp. Med. Biol..

[14] Zachow S, Lamecker H, Elsholtz B, Stiller M (2005). Reconstruction of mandibular dysplasia using a statistical 3D shape model. Comput. Assist. Radiol. Surg. (CARS).

[15] Wang E, Tran KL, Dheygere E, Prisman E (2021). Predicting the premorbid shape of a diseased mandible. Laryngoscope.

[16] Gillingham RL, Mutsvangwa TEM, van der Merwe J (2023). Reconstruction of the mandible from partial inputs for virtual surgery planning. Med. Eng. Phys..

[17] Gunz P, Mitteroecker P, Neubauer S, Weber GW, Bookstein FL (2009). Principles for the virtual reconstruction of hominin crania. J. Hum. Evol..

[18] Cardini A, Elton S (2007). Sample size and sampling error in geometric morphometric studies of size and shape. Zoomorphology.

[19] Cardini A, Seetah K, Barker G (2015). How many specimens do I need? Sampling error in geometric morphometrics: Testing the sensitivity of means and variances in simple randomized selection experiments. Zoomorphology.

[20] Liao P, Chen T, Chung PC (2001). A fast algorithm for multilevel thresholding. J. Inf. Sci. Eng..

[21] Lorensen WE, Cline HE (1987). Marching cubes: A high resolution 3D surface construction algorithm. SIGGRAPH Comput. Graph..

[22] Botsch, M. & Kobbelt, L. A remeshing approach to multiresolution modelling. In *Proceedings of the 2004 Eurographics/ACM SIGGRAPH Symposium on Geometry Processing (SGP '04)* 185–192 (Association for Computing Machinery, 2004).

[23] Coquerelle M (2010). The association between dental mineralization and mandibular form: A study combining additive conjoint measurement and geometric morphometrics. J. Anthropol. Sci..

[24] Bardua C, Felice RN, Watanabe A, Fabre AC, Goswami A (2019). A practical guide to sliding and surface semilandmarks in morphometric analyses. Integr. Org. Biol..

[25] Bookstein FL (1997). Landmark methods for forms without landmarks: Morphometrics of group differences in outline shape. Med. Image Anal..

[26] Gunz P, Mitteroecker P (2013). Semilandmarks: A method for quantifying curves and surfaces. Hystrix It. J. Mamm..

[27] Gunz, P., Mitteroecker, P. & Bookstein, F. L. Semilandmarks in three dimensions. In *Modern Morphometrics in Physical Anthropology* (ed. Slice, D. E.) 73–98 (Kluwer Academic/Plenum Publishers, 2005).

[28] Rohlf FJ, Slice D (1990). Extensions of the Procrustes method for the optimal superimposition of landmarks. Syst. Zool..

[29] Kemkes-Grottenthaler A, Löbig F, Stock F (2002). Mandibular ramus flexure and gonial eversion as morphologic indicators of sex. Homo.

[30] Loth SR, Henneberg M (1996). Mandibular ramus flexure: A new morphologic indicator of sexual dimorphism in the human skeleton. Am. J. Phys. Anthropol..

[31] Klingenberg CP (2011). MorphoJ: An integrated software package for geometric morphometrics. Mol. Ecol. Resour..

[32] Fruciano C (2016). Measurement error in geometric morphometrics. Dev. Genes Evol..

[33] Peres-Neto PR, Jackson DA, Somers KM (2005). How many principal components? Stopping rules for determining the number of non-trivial axes revisited. Comput. Stat. Data Anal..

[34] Hammer, Ø., Harper, D. A. T. & Ryan, P. D. PAST: Paleontological statistics software package for education and data analysis. *Palaeontologia Electronica* http://palaeo-electronica.org/2001_1/past/issue1_01.htm (2001).

[35] Porto A, Rolfe S, Maga AM (2021). ALPACA: A fast and accurate computer vision approach for automated landmarking of three-dimensional biological structures. Methods Ecol. Evol..

[36] Fournier G, Maret D, Telmon N, Savall F (2023). An automated landmark method to describe geometric changes in the human mandible during growth. Arch. Oral Biol..

[37] Verhelst PJ (2021). Automatic 3D dense phenotyping provides reliable and accurate shape quantification of the human mandible. Sci. Rep..

[38] White JD (2019). MeshMonk: Open-source large-scale intensive 3D phenotyping. Sci. Rep..

[39] Kim SG (2012). Development of 3D statistical mandible models for cephalometric measurements. Imaging Sci. Dent..

[40] Bookstein FL (1991). Morphometric Tools for Landmark Data: Geometry and Biology.

[41] Gower JC (1975). Generalized Procrustes analysis. Psychometrika.

[42] Bermejo E (2021). Automatic landmark annotation in 3D surface scans of skulls: Methodological proposal and reliability study. Comput. Methods Programs Biomed..

[43] Franklin D, Oxnard CE, O'Higgins P, Dadour I (2007). Sexual dimorphism in the subadult mandible: Quantification using geometric morphometrics. J. Forensic Sci..

[44] Bosman AM, Moisik SR, Dediu D, Waters-Rist A (2017). Talking heads: Morphological variation in the human mandible over the last 500 years in the Netherlands. Homo.

[45] Guevara-Perez SV, De-la-Rosa-Castolo G, Thollon L, Behr M (2018). A 3D characterization method of geometric variation in edentulous mandibles. Morphologie.

[46] Bergmann I, Hublin JJ, Gunz P, Freidline SE (2021). How did modern morphology evolve in the human mandible? The relationship between static adult allometry and mandibular variability in Homo sapiens. J. Hum. Evol..

[47] Lagravère MO (2010). Intraexaminer and interexaminer reliabilities of landmark identification on digitized lateral cephalograms and formatted 3-dimensional cone-beam computerized tomography images. Am. J. Orthod. Dentofacial Orthop..

[48] Park J (2019). Reliability of 3D dental and skeletal landmarks on CBCT images. Angle Orthod..

[49] Kim JH, An S, Hwang DM (2022). Reliability of cephalometric landmark identification on three-dimensional computed tomographic images. Br. J. Oral Maxillofac. Surg..

[50] Mitchell DR, Kirchhoff CA, Cooke SB, Terhune CE (2021). Bolstering geometric morphometrics sample sizes with damaged and pathologic specimens: Is near enough good enough?. J. Anat..

[51] Khramtsova EA, Davis LK, Stranger BE (2019). The role of sex in the genomics of human complex traits. Nat. Rev. Genet..

[52] Aneja D, Vora SR, Camci ED, Shapiro LG, Cox TC (2015). Automated detection of 3D landmarks for the elimination of non-biological variation in geometric morphometric analyses. Proc IEEE Int. Symp. Comput. Based Med. Syst..

[53] Costa Mendes L (2021). Sexual dimorphism of the mandibular conformational changes in aging human adults: A multislice computed tomographic study by geometric morphometrics. PLoS One.

[54] Hutchinson EF, Farella M, Kramer B (2015). Importance of teeth in maintaining the morphology of the adult mandible in humans. Eur. J. Oral Sci..

[55] Zorba E, Moraitis K, Eliopoulos C, Spiliopoulou C (2012). Sex determination in modern Greeks using diagonal measurements of molar teeth. Forensic Sci. Int..

[56] Capitaneanu C, Willems G, Thevissen P (2017). A systematic review of odontological sex estimation methods. J. Forensic Odontostomatol..

[57] Vallabh R, Zhang J, Fernandez J, Dimitroulis G, Ackland DC (2020). The morphology of the human mandible: A computational modelling study. Biomech. Model Mechanobiol..

[58] Kranioti EF, Gomez-García-Donas J, Langstaff H (2014). Sex estimation of the Greek mandible with the aid of discriminant function analysis and posterior probabilities. Rom. J. Legal Med..

[59] Oettlé AC, Pretorius E, Steyn M (2009). Geometric morphometric analysis of the use of mandibular gonial eversion in sex determination. Homo.

[60] Balci Y, Yavuz MF, Cağdir S (2005). Predictive accuracy of sexing the mandible by ramus flexure. Homo.

[61] Premkumar A (2023). Sex determination using mandibular ramus flexure in South Indian population—a retrospective study. J. Forensic Odontostomatol..

[62] Giles E (1964). Sex determination by discriminant function analysis of the mandible. Am. J. Phys. Anthropol..

[63] Steyn M, Işcan MY (1998). Sexual dimorphism in the crania and mandibles of South African whites. Forensic Sci. Int..

[64] Kharoshah MA, Almadani O, Ghaleb SS, Zaki MK, Fattah YA (2010). Sexual dimorphism of the mandible in a modern Egyptian population. J. Forensic Leg. Med..

[65] Dong H (2015). Sexual dimorphism of the mandible in a contemporary Chinese Han population. Forensic Sci. Int..

[66] Berg GE, Kenyhercz MW (2017). Introducing Human Mandible Identification [(hu)MANid]: A free, web-based GUI to classify human mandibles. J. Forensic Sci..

[67] Tunis TS (2017). Sex estimation using computed tomography of the mandible. Int. J. Legal Med..

[68] Okkesim A, Sezen-Erhamza T (2020). Assessment of mandibular ramus for sex determination: Retrospective study. J. Oral Biol. Craniofac. Res..

[69] Farhi M (2023). Evaluation of the (hu)MANid program for sex and ancestry estimation in a diverse, contemporary CT scan-based sample. J. Forensic Sci..

[70] Franklin D, O'Higgins P, Oxnard CE, Dadour I (2006). Determination of sex in South African blacks by discriminant function analysis of mandibular linear dimensions: A preliminary investigation using the Zulu local population. Forensic Sci. Med. Pathol..

