# Supplementary material for: Shape variation and sex differences of the adult human mandible evaluated by geometric morphometrics
Source: Sci Rep. 2024 Apr 12;14:8546. doi: 10.1038/s41598-024-57617-7 (PMC11014969; doi:10.1038/s41598-024-57617-7)

Shape variation and sex differences of the adult human mandible evaluated by geometric morphometrics.

Supplementary material

Authors

Aspasia Chalazoniti[1]
ORCID: 0000-0002-9833-1494
Email: ahal@dhal.com

Wanda Lattanzi[2, 3]
ORCID: 0000-0003-3092-4936
Email: Wanda.Lattanzi@unicatt.it

Demetrios J. Halazonetis[4]*
ORCID: 0000-0002-6501-8709
Email: dhal@dhal.com; dhalaz@dent.uoa.gr
*Corresponding author

1. Department of Prosthodontics, School of Dentistry, National and Kapodistrian University of Athens, Greece.
2. Department of Life Science and Public Health, Università Cattolica del Sacro Cuore, Rome, Italy.
3. Unit of Paediatric Neurosurgery, Fondazione Policlinico Universitario A. Gemelli IRCCS, Rome, Italy.
4. Department of Orthodontics, School of Dentistry, National and Kapodistrian University of Athens, Greece.

Supplementary Table S1. List of landmarks and curves. R: right, L: left. Total number of fixed landmarks: 9. Total number of semilandmarks: 510.

Curves	
Notch (R & L)	From the tip of the coronoid to the anterior surface of the condyle.
Coronoid (R & L)	From the tip of the coronoid, along the anterior border of the ramus, to the level of the dentition.
Posterior inferior	From the posterior surface of the right condyle, along the posterior border of the ramus, past Gonion, along the inferior border of the mandible, to the symphysis, and similarly on the left side.
Symphysis	From the labial alveolar crest (infradentale), inferiorly around the symphysis, and up to the lingual alveolar crest (linguale).
Alveolar buccal	Along the alveolar buccal crest, from the midpoint of the second molar of one side to the corresponding point of the other side. In case of a missing molar, the position was estimated from the molar of the opposite side, or was estimated from the position of the remaining teeth.
Alveolar lingual	Similar to Alveolar buccal, but on the lingual side.
Landmarks (n = 9)	
Condyle lateral (R & L)	The lateral pole of the condyle.
Condyle medial (R & L)	The medial pole of the condyle.
Coronoid (R & L)	The tip of the coronoid process.
Gonion (R & L)	The most posterior and inferior point at the gonial angle. Located as the farthest point of the gonial angle from the line connecting the condyle and Gnathion.
Gnathion	The most inferior and anterior point of the symphysis, on the midsagittal plane. Located as the farthest point from the condyle.
Semilandmarks sliding on curves (n = 84)	
Notch	5 semilandmarks on each of the Notch curves
Coronoid	5 semilandmarks on each of the Coronoid curves
Posterior inferior	31 semilandmarks on the Posterior inferior curve
Alveolar buccal	12 semilandmarks on the Alveolar buccal curve
Alveolar lingual	10 semilandmarks on the Alveolar lingual curve
Symphysis	11 semilandmarks on the Symphysis curve
Semilandmarks sliding on the mesh surface (n = 426)	
Exterior surface	100 semilandmarks on the right side and 100 semilandmarks on the left side, on the buccal/labial surface of the mandible.
Interior surface	80 semilandmarks on the right side and 80 semilandmarks on the left side, on the lingual surface of the mandible.
Condyle Coronoid medial	26 semilandmarks on the right side and 26 semilandmarks on the left side, on the medial surface of the condylar and coronoid processes.
Condylar head	7 semilandmarks on each condylar head.

Supplementary Table S2. Procrustes ANOVA for evaluating measurement error of shape.

Effect	Sum of Squares	Mean Squares	df	F	P
Individual	0.13117082	4.454×10^{-6}	29450	239.50	< 0.0001
Repeat	0.00057650	1.86×10^{-8}	31000		

Supplementary Table S3. Percent variance and cumulative variance of mandibular shape described by each principal component, in shape space and form space, for the whole sample. Only PCs describing at least 1% variance, and any PC considered non-trivial, as determined by both Avg-Rnd and Rnd-Lambda (marked with an asterisk), are listed.

	Shape space		Form Space	
PC	Percent variance	Percent cumulative variance	Percent variance	Percent cumulative variance
PC 1	20.2 *	20.2	47.1 *	47.1
PC 2	17.4 *	37.6	11.2 *	58.2
PC 3	11.4 *	49.0	9.2 *	67.4
PC 4	7.7 *	56.7	5.2 *	72.6
PC 5	5.3 *	62.1	3.8 *	76.4
PC 6	4.4 *	66.4	2.9 *	79.3
PC 7	3.4 *	69.8	2.3 *	81.7
PC 8	2.6 *	72.4	1.9 *	83.6
PC 9	2.5 *	74.9	1.4 *	85.0
PC 10	2.2 *	77.1	1.3 *	86.3
PC 11	1.8 *	78.9	1.2 *	87.5
PC 12	1.6 *	80.4	1.0 *	88.5
PC 13	1.4	81.9	0.9 *	89.3
PC 14	1.3	83.2		
PC 15	1.1	84.3		
PC 16	1.0	85.3		

Supplementary Table S4. Percent correct sex classification based on LDA and number of included PCs (non-trivial PCs only).

PCs	Shape space	Form Space
PC 1-2	59%	91%
PC 1-3	72%	92%
PC 1-4	72%	93%
PC 1-5	72%	93%
PC 1-6	69%	93%
PC 1-7	70%	93%
PC 1-8	70%	91%
PC 1-9	68%	92%
PC 1-10	66%	92%
PC 1-11	72%	92%
PC 1-12	72%	92%
PC 1-13	-	92%

Supplementary Figure S1. The template of curves and landmarks, overlaid on a symmetric simplified mesh, expanded by 1.5 mm (transparent overlay) to ensure correct landmark projection when transferring points, mainly those on the medial and lateral ramus surfaces. Semilandmarks are coloured differently, based on their position, to easily detect any such projection errors.

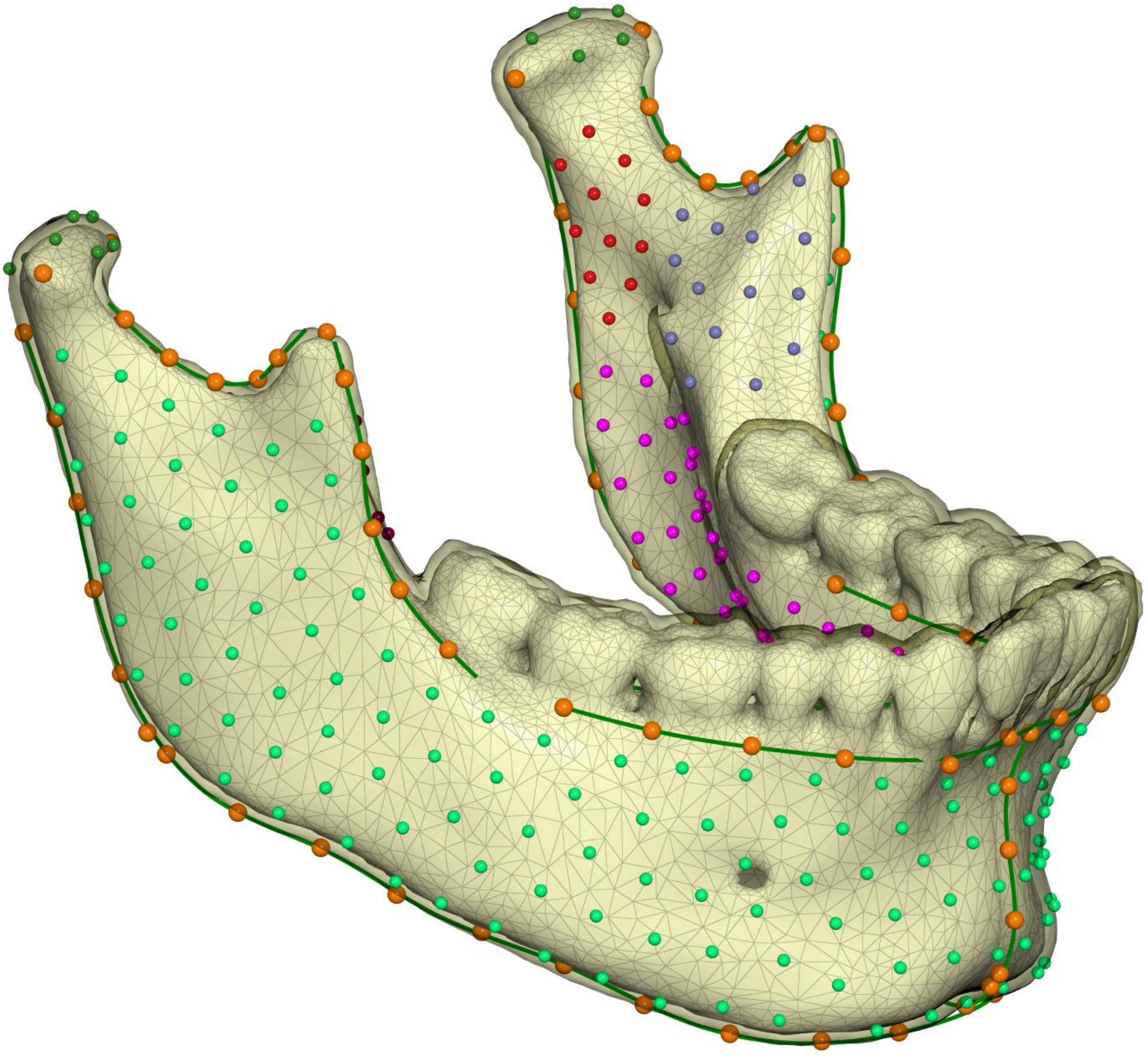

Supplementary Figure S2. Iterative procedure for locating condylar poles (here shown schematically in 2D). **a**: initial placement of landmarks and condylar axis. **b**: landmarks are repositioned outwards along the condylar axis. **c**: landmarks are projected on the condylar surface at the closest point (dotted lines: tangents to surface). **d**: new condylar axis.

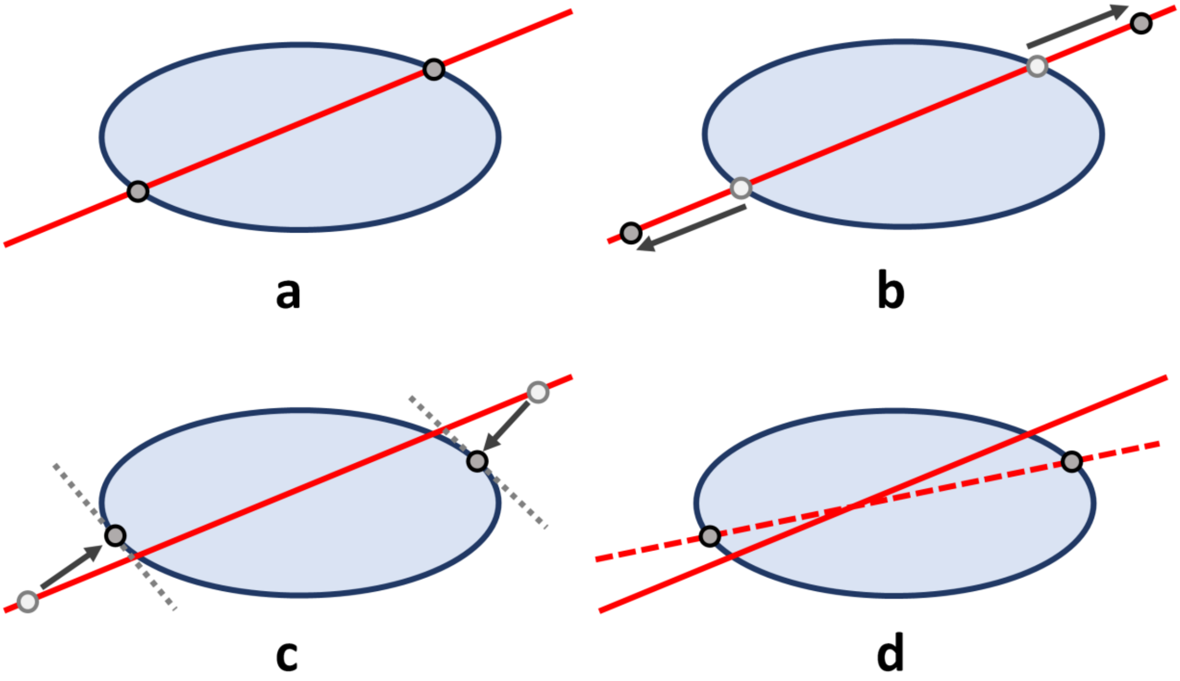

Supplementary Figure S3. Landmarks included for the ramus analysis are shown in the blue region.

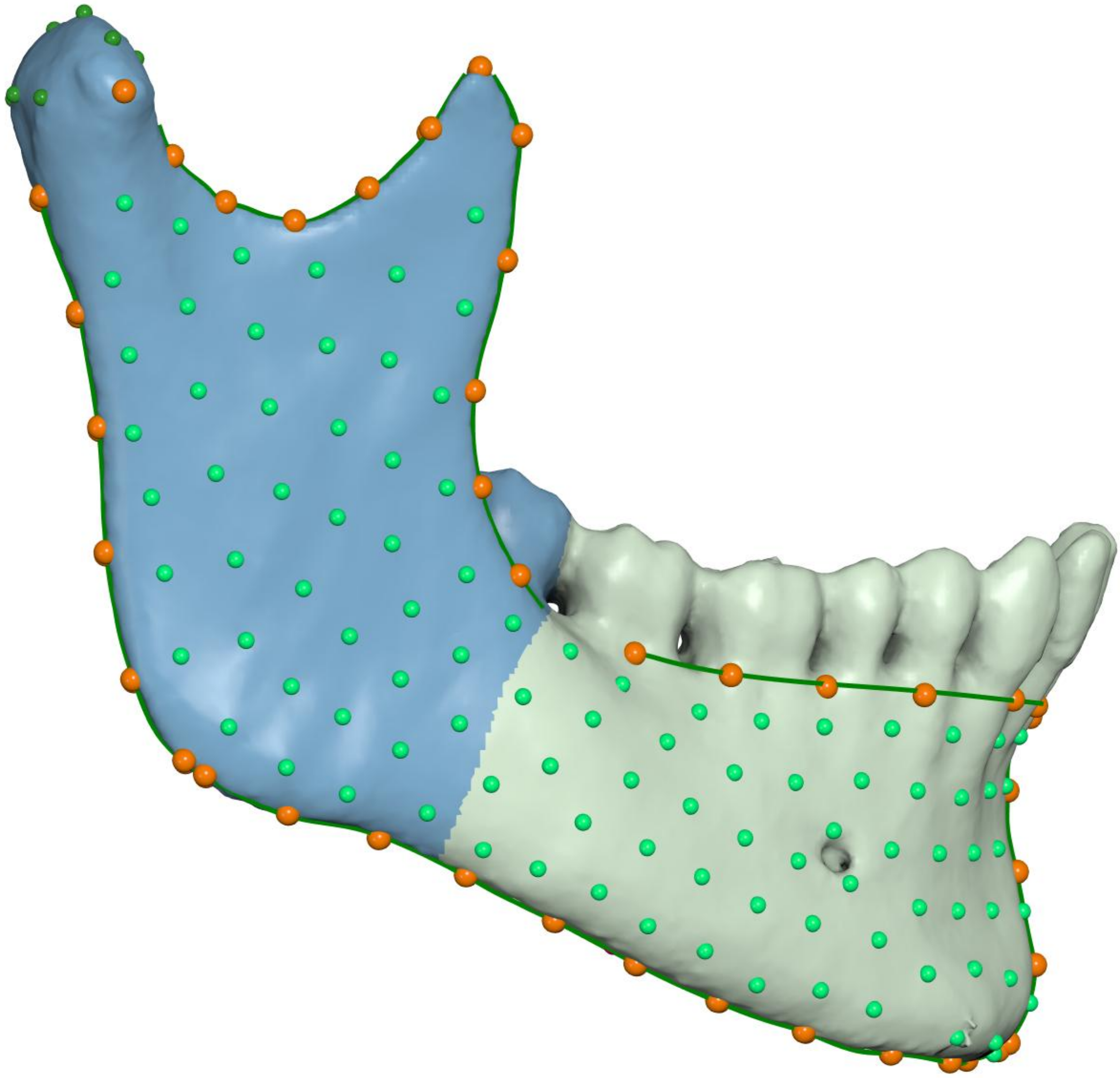

Supplementary Figure S4. Repeated digitization (filled triangles) in shape space, showing small error relative to the original digitization (filled circles) and overall sample variability. Open circles: non-repeats. Blue: male, red: female.

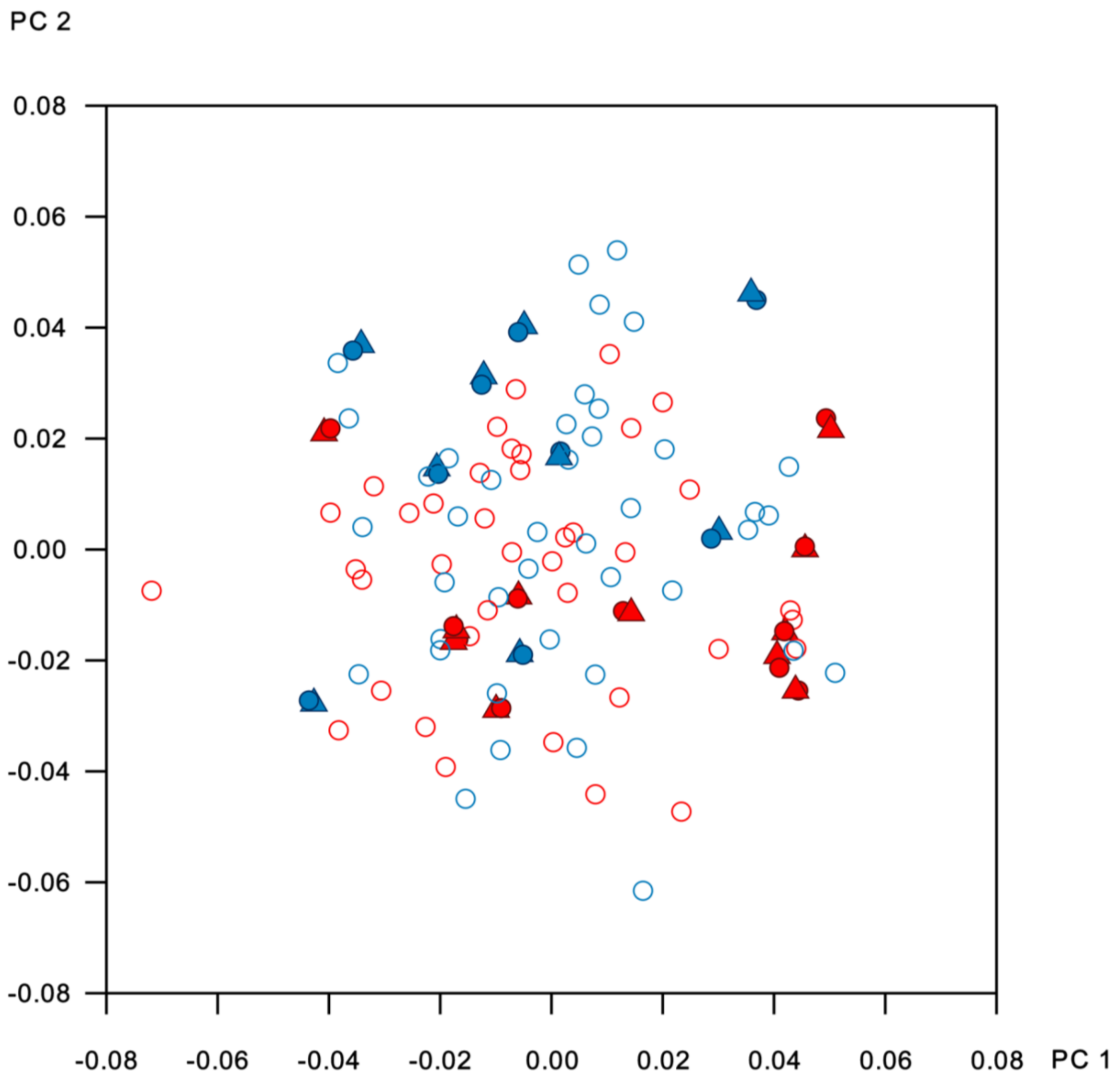

Supplementary Figure S5. Plot of the sample in form space. Blue: males, red: females. Black line: vector of pure size variance.

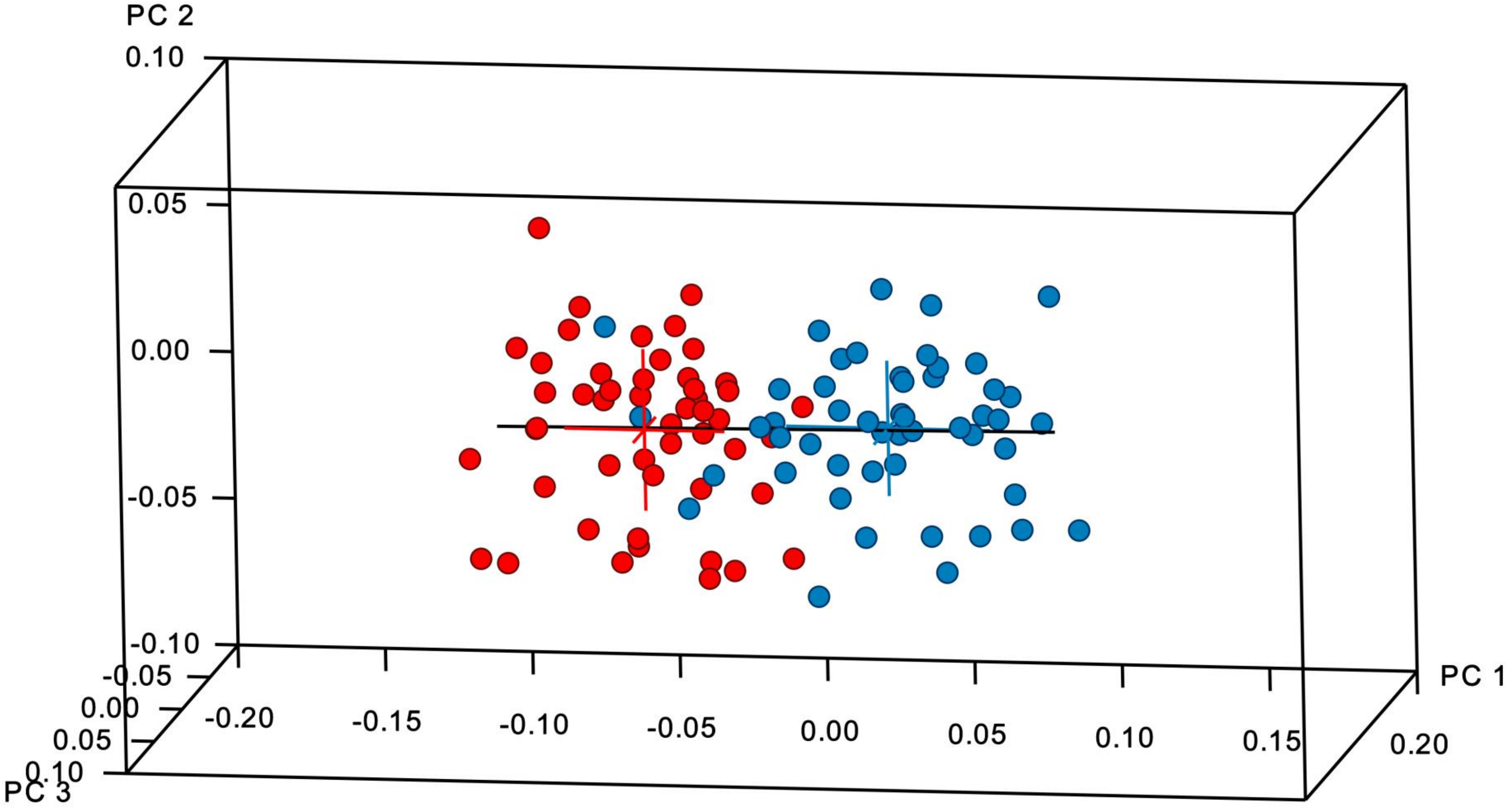

Supplementary Figure S6. Superimposition of mandibular shape warped to ±3 standard deviations along the regression vector of shape on number of missing teeth for the pooled sample. Green: dentate; purple: edentulous. Here, the same exemplar shape has been warped, so teeth are visible in both extremes.

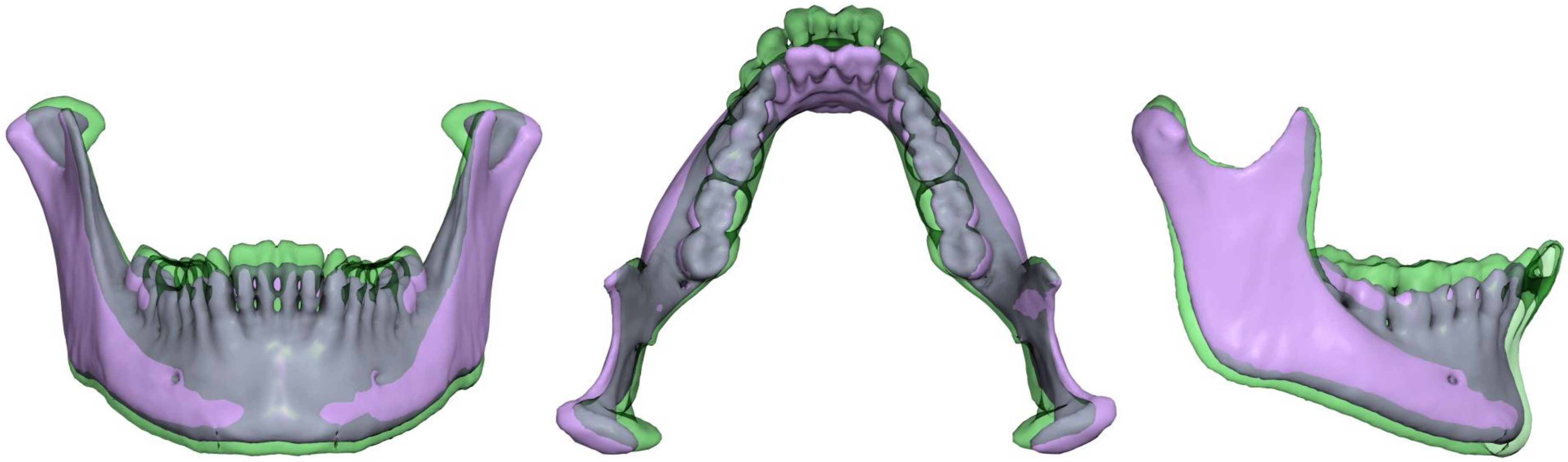

Supplement: Supplementary file 1 — Supplementary Information. [file 41598_2024_57617_MOESM1_ESM.pdf]
